# Allele-specific real-time PCR testing for minor macrolide-resistant *Mycoplasma Pneumoniae*

**DOI:** 10.1186/s12879-019-4228-4

**Published:** 2019-07-12

**Authors:** Dongxing Guo, Wenjuan Hu, Baoping Xu, Jingyi Li, Dan Li, Shaogang Li, Zhaoyong Wu, Ran Wei, Xiujun Tian, Kunling Shen, Deli Xin

**Affiliations:** 1 Tropical Medicine Research Institute, Beijing Friendship Hospital, Capital Medical University, No. 95 Yong an Road, Xicheng District, Beijing, China; 2 Department of Paediatrics, Civil Aviation General Hospital, Beijing, China; 3 Department of Respiratory, Beijng Children’s Hospital, Capital Medical University, No. 56 South Lishi Road, Xicheng District, Beijing, China

**Keywords:** *Mycoplasma pneumoniae*, Allele-specific real-time PCR, 23S rRNA, A2063G, A2064G

## Abstract

**Background:**

The point mutations in 23S rRNA gene of *Mycoplasma pneumoniae* (*M. pneumoniae*) can lead to high-level resistance to macrolides. This study aimed to evaluate allele-specific real-time PCR (ASPCR) to detect the resistance-related mutations located at positions A2063G and A2064G of 23S rRNA gene.

**Methods:**

We detected 178 pharyngeal swab specimens and calculated the proportions of resistant and sensitive quasispecies using ASPCR assays. ASPCR assays can detect down to 10 copies of 23S rRNA gene and achieved sensitivities of < 0.1% for A2063G and A2064G. We also compared the findings of ASPCR with the results of nested PCR with sequencing.

**Results:**

Of 178 samples, 164 were found to have *M. pneumoniae* including 90.85% (149/164) samples with macrolide-resistant *M. pneumoniae* (MRMP) quasispecies by ASPCR, while 153 were found to be *M. pneumoniae*-positive including 71.90% (110/153) samples with MRMP quasispecies by nested PCR with sequencing. Of the 164 *M. pneumoniae-*positive samples, 61.59% (101/164) had the mixed population of wild-type and mutant *M. pneumoniae*, and 56.44% (57/101) of the latter contained the mutations at low frequency (≤50%).

**Conclusion:**

ASPCR indicated that sensitive and resistant quasispecies coexisted in most of the *M. pneumoniae* positive samples. The ASPCR was a highly sensitive, accurate and rapid method for detecting the macrolide resistance-associated mutations and it could provide earlier and more drug-resistant information for *M. pneumoniae* research and the clinical therapy.

## Background

*Mycoplasma pneumoniae* (*M. pneumoniae*) can cause atypical pneumoniae and many respiratory illnesses, particularly in childhood and specify young adults, its infection rate range from 10% to.

80% [1–3]. Macrolides and related antibiotics have been generally considered to be the first-choice antibiotic for the treatment of *M. pneumoniae* infection [4]. However, since the first isolates macrolide-resistant *M. pneumoniae* (MRMP) in 2001 [5], MRMP has been spreading globally for about seventeen years, with prevalences ranging from below 10% in Europe [1, 6–10], approximately 30% in Israel [11], and up to 90% in Asia [12–14].

The commonly used phenotypic methods for determining susceptibility to macrolides is to measure the minimum inhibitory concentration (MIC), and this process is rather time-consuming.

In previous studies, point mutations in several positions have been reported to be related to development of macrolide resistance in *M. pneumoniae* [15]. Among them, the 2063 and 2064 point mutations in the peptidyl-transferase loop of domain V of 23S rRNA, which interferes with the binding of macrolides to rRNA [1, 5, 16, 17], are the major mutations responsible for different grade macrolide-resistance of *M. pneumoniae* [5, 17]. For example, one study reported that 90.90% had an A2063G transition and 9.10% had an A2064G transition in domain V of the 23S rRNA gene among 55 macrolide-resistant strains [18]. Other mutations, such as those at positions 2617, 2067 and 2611 in domain V of the single-copy 23S rRNA gene, and mutations in the ribosomal proteins L4 and L22, are very rare [19, 20]. Several molecular detection methods for identification of these mutations include sequencing of PCR products, real-time PCR, pyrosequencing, restriction fragment length polymorphism (RFLP) analysis, high-resolution melting curve analysis and allele-specific PCR [21–26]. However, most methods have limitations as following: sequencing of PCR products and RFLP analysis are time-consuming and expensive, and have no value in clinical practice due to only used in research; real-time PCR may be much easier, quicker, and simpler to perform than other methods, but may not be able to distinguish between the 2063 and the 2064 mutation without additional simplex real-time PCR, and cannot detect unknown mutations [8, 19]. Therefore, a rapid, sensitive, and specific laboratory test is vital for rapid detection of *M. pneumoniae* infections.

Allele-specific real-time PCR (ASPCR) applies the real-time PCR to allele-specific PCR and integrates the advantages of these two systems. ASPCR has increased the sensitivity of the allele-specific PCR several-fold and quantified the PCR products [27, 28]. Therefore, ASPCR is a highly sensitive and time-saving method for detection of point mutations and is significantly labor-intensive and reproducible. In this study, we developed ASPCR assays for detecting 23S rRNA gene of *M. pneumoniae* and determine the macrolide resistance-associated mutations at 2063 (A2063G) and 2064 (A2064G) sites. In addition, we detected 178 pharyngeal swab specimens using ASPCR to reveal the prevalence of macrolide-resistant and sensitive *M. pneumoniae* quasispecies in clinical specimens.

## Methods

### Specimens and reference strains

A total of 178 pharyngeal swab specimens were tested using ASPCR. All specimens were collected from pediatric patients (aged, 2–12 years) with a clinical diagnosis of *M. pneumoniae* infection from August 2013 to March 2015. The reference strain M129 (ATCC 29342) used in this study was preserved in our laboratory. The specificity of the ASPCR was tested using DNA of the following reference strains: *Neisseria mucosa, Klebsiella pneumoniae, Escherichia coli, Staphylococcus aureus, Streptococcus pneumoniae, Pseudomonas aeruginosa, Mycoplasma hominis, Mycoplasma fermentans, Mycoplasma pyriformis and Ureaplasma urealyticum*. All reference strains were preserved in our laboratory.

### Primer design

All the primers were designed based on the sequence of *M. pneumoniae* reference strain M129 (GenBank accession no. U00089). The mutant-specific primers (Msp) and non-specific primers (Np) were designed to determine the macrolide resistance-associated mutations at 2063 (A2063G) and 2064 (A2064G) site. The mutant-specific primers incorporate the target mutation in their 3′-end and plus three intentional mismatch bases (hypoxanthylic acid) near the end to enhance the specificity of mutant-specific amplification (Table 1). The non-specific primer was identical to the corresponding mutant-specific primer, except that the sequence ends right before the mutation position and without the substitutions of hypoxanthylic acid. The same reverse primer was used in the mutant-specific and the non-specific reactions performed in separate wells.

**Table 1:** Oligonucleotide sequences and locations on the M129 genome

Name	Sequence (5′-3′)	Position in M129
23S rRNA-F	CTTTCTAATGGAGTTTTTTACTT	119,806—119,828
23S rRNA-R	GCTTGGTGCTTTCCTATTCT	123,068—123,087
23SA2063G-F	GGACGG**G**AAGACCCCGTGAAGCTTTACT	122,094—122,121
23SA2064G-F	GGACGGA**G**AGACCCCGTGAAGCTTTACT	122,094—122,121
23S2063/2064-R	CGTTGCGCCTAACGGGTGTCTTCAC	122,069—122,093
NU-F (Np)	TTAGGCGCAACGGGACGG	122,082—122,099
2063MU-F (Msp)	TTAGGCGCAACGGGA***III*G**	122,082—122,100
2064MU-F (Msp)	TTAGGCGCAACGGGA***III***A**G**	122,082—122,101
23S2063/4D-R	CTGGATAACAGTTACCAATTAGAACAGC	122,233—122,260

The target mutations in the primers of sit-directed mutations (23SA2063G-F and 23SA2064G-F) are shown in boldface. The target mutations at the end of the mutant-specific primers (Msp) are shown in boldface and underlined. The internal mismatches in the mutant-specific primers (Msp) are shown in boldface italics

### Construction of standards and ASPCR amplification

The standards of the ASPCR assays for A2063G and A2064G were then constructed. Plasmids containing a wild-type fragment of *M. pneumoniae* 23S rRNA full-length sequence were obtained by cloning the PCR products amplified by the primers 23SrRNA-F and 23SrRNA-R into pMD18-T (pMD™18-T Vector Cloning Kit, TaKaRa, Japan) vectors. The mutations of A2063G and A2064G were introduced into wild-type plasmids by sit-directed mutagenesis (TaKaRa MutanBEST Kit, TaKaRa, Japan) using primers 23SA2063G-F, 23SA2064G-F, and 23S2063/2064-R. The plasmids containing mutations were confirmed by sequencing and quantified by spectrophotometer. Serial 10-folds dilutions of plasmids ranging from 10 to 10^6^ copies/μL were made as standards for ASPCR.

The mutant-specific and nonspecific standard curves of A2063G and A2064G were obtained by amplifying the mutant standards with the corresponding primer sets. The ASPCR mix contained 12.5 μL power SYBR Green PCR Master 2 × Mix, 2 μL mutant-specific/non-specific upstream primer, 2 μL corresponding downstream-primer, 2 μL DNA templates, 6.5 μL ddH_2_O. The reaction conditions were 50 °C for 2 min, followed by a denaturation step at 95 °C for 10 min, 40 cycles of real-time PCR amplification (95 °C for 15 s, 60 °C for 1 min) with a final dissociation stage (95 °C for 15 s, 60 °C for 30 s, 95 °C for 15 s). Each reaction was conducted in triplicate.

### Evaluation of ASPCR

The threshold cycle (Ct) values of 10^7^ copies/μL of mutant and wild-type plasmids with the specific primer were tested and calculated the ΔCt. The mixture templates were generated by adding 10^5^ copies of wild-type DNA to serial dilutions (10^6^–10^1^ copies) of mutant DNA. We compared the Ct values of the mutant template and mixture of mutant and wild-type plasmids to evaluate the discrimination ability of ASPCR.

The mixture templates in which the proportion of mutant plasmids ranged from 0.01 to 100% were generated by adding 10^5^ copies of wild-type DNA into the serial dilutions (10^6^–10^1^ copies) of mutant DNA. The cut-off value was defined as the mean Ct value plus three standard deviations (SD) of 12 independent determinations of 10^5^ copies wild-type template with mutant-specific primer. Mutants were inferred to be present in the mixing templates when the Ct value was less than the cutoff value. The measured proportions and nominal proportions of the mixtures with mutant template ranging from 0.01 to 100% were compared to evaluate the accuracy of ASPCR. The sensitivity of ASPCR was determined by the cutoff value and accuracy. The coefficient of variations (CVs) of intra-assay and inter-assay were calculated to evaluate the reproducibility of ASPCR.

### Detection of clinical specimens

Genomic DNA was extracted from M129 strain-enriched culture solution and pharyngeal swab specimens using the QIAamp® DNA Mini Kit (QIAGEN, Shanghai, Germany), according to the manufacturer’s protocol. The DNA fragment in domain V of *M. pneumoniae* 23S rRNA region were detected using nested PCR method as previously reported [29, 30] and ASPCR as above. Each sample was amplified by mutant-specific and non-specific primers of each mutation separately and the amplifications ran in duplicate. The corresponding standards of the mutations were tested in each plate, and the standard cure was drawn every time for quantification. The Ct values of the amplifications of clinical samples using specific and non-specific primer were interpolated into the corresponding standard curves to get the numbers of mutant DNA and the total DNA. Then the proportions of A2063G and A2064G MRMP quasispecies populations were calculated.

### Statistical analysis

All statistical analysis was performed using IBM SPSS Statistics 19. Continuous variables were compared with the Student’s t test or Analysis of Variance, and categorical variables were compared with the Chi-square test. To evaluate the coincidence ratio between the ASPCR and nested PCR assay, the kappa coefficient was calculated using Chi-square test. A *P*-value of < 0.05 were considered to statistical significance.

## Results

### Standard curve and amplification efficiency

**Fig. 1:**
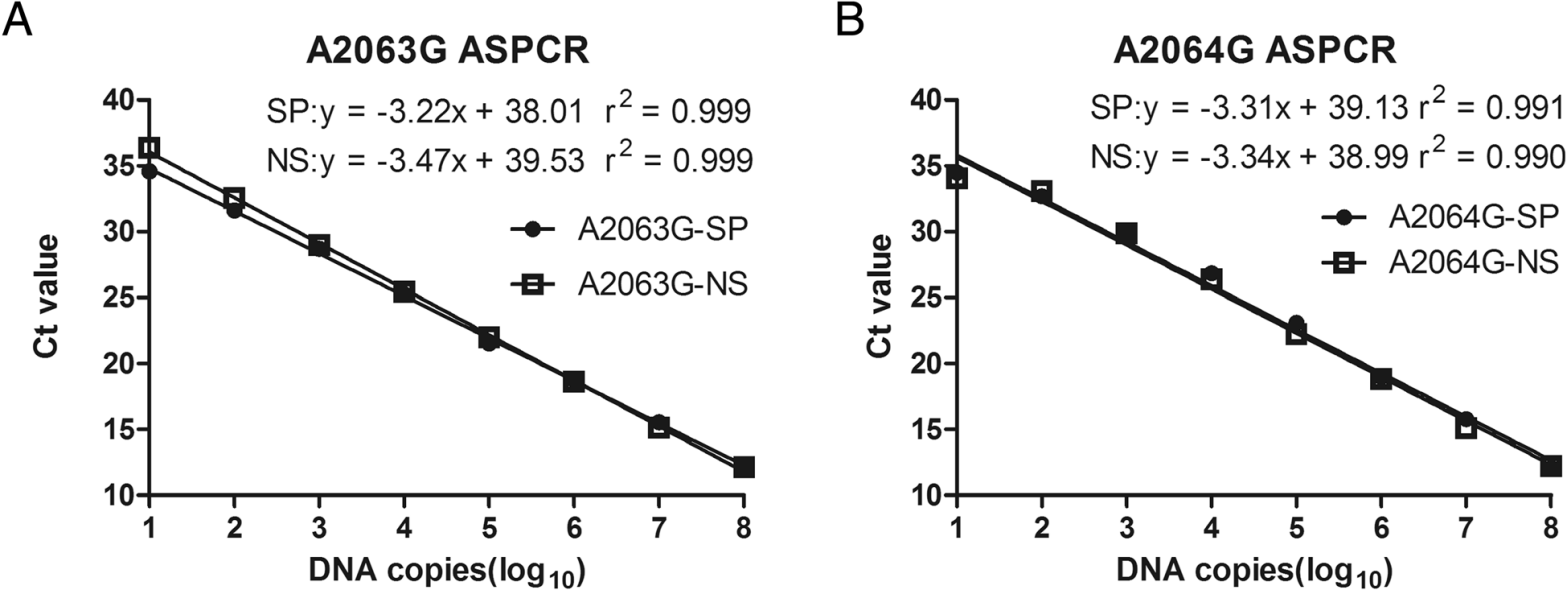
Specific (SP, solid symbols) and non-specific (NS, open squares) standard curves of A2063G and A2064G in 23Sr RNA gene of *M. pneumoniae*. **A**: A2063G standard curve. **B:** A2064G standard curve. SP, specific primer; NP, non-specific primer

The assays could detect the 23S rRNA gene down to 10 copies/reaction and the Ct values were log-linearly correlated with the copy numbers of the standards over the range of 10^1^–10^8^ copies **(**Fig. 1**)** for each set of mutant-specific and non-specific primers. The mutant-specific and non-specific amplification efficiencies of each set of primers were comparable. The correlation coefficients (r^2^) of all primer sets on their respective corresponding standards were higher than 0.99. These standard curves could be used to determine the copy number of each sample. Lastly, it was worth mentioning that no fragments were amplified in the negative controls (no template control (NTC)) in each PCR reaction.

### Specificity of ASPCR

**Fig. 2:**
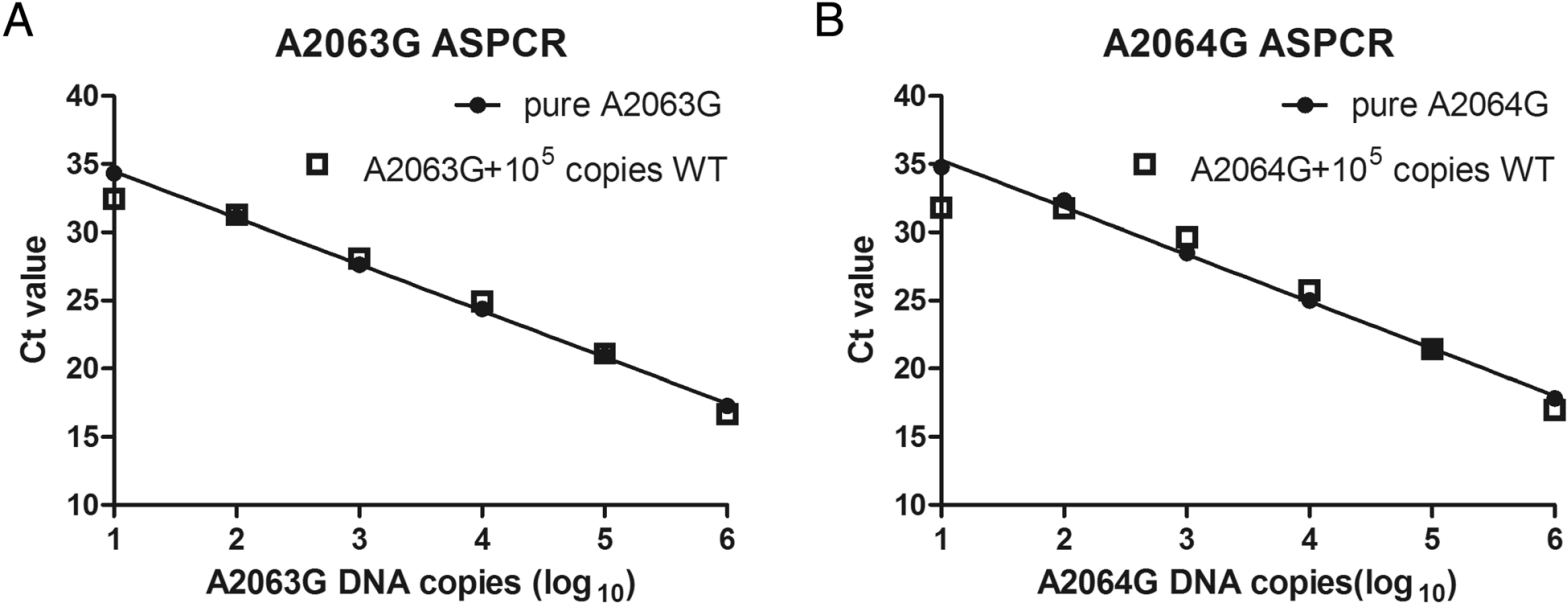
Allelic discrimination of the ASPCR assays. **A**: ASPCR assays of A2063G. **B**: ASPCR assays of A2064G. Ct value comparisons of the serial dilutions (10^6^–10^1^ copies) of mutant DNA with 10^5^ copies of wild-type DNA (open squares) and the mutant DNA without the addition of wild-type DNA (solid symbols). The addition of non-complementary wild-type templates to the mutant standard did not significantly alter the Ct values until the mutant DNA was 10 copies in the reaction. The Ct value of 10^5^ copies of wild-type WT template amplified by A2063G and A2064G specific primers was 32.78 ± 0.26 and 31.75 ± 0.34, respectively

To evaluate the specificities of the mutant-specific primers, we compared the Ct values of identical amounts of mutant and wild-type DNA with the corresponding mutant-specific primer set. The ΔCt value represented the differences in Ct values when 10^7^ copies mutant and wild-type plasmids were amplified with corresponding mutant-specific primers of each mutation. For the mutant-specific primer sets of A2063G and A2064G, the ΔCt values were 16.99 and 10.56, respectively, all ΔCt values were > 10. These results indicated that the amplification efficiency dramatically decreased for wild-type template using mutant-specific primer and all the specific primer sets could successfully discriminate the mutant template from wild-type template. In the second experiment to evaluate the discriminatory ability of each assay, the Ct values of mutant DNA and the mixture of mutant and wild-type plasmids were linearly correlated (Fig. 2). These results illustrated that the discriminatory ability of the two ASPCR assays remained unaltered until the mutant DNA was less than 0.01%.

**Fig. 3:**
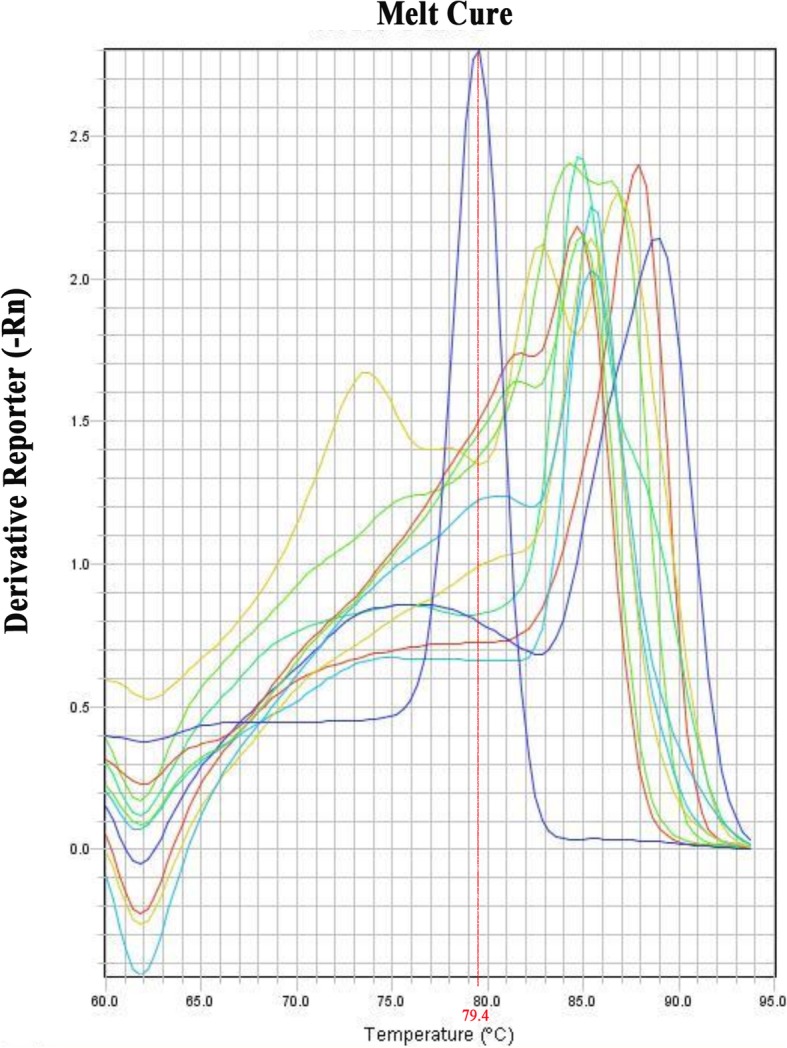
The amplification plot and melting curves for *M. pneumoniae* and the reference strains

In addition, the specificity of ASPCR for *M. pneumoniae* was tested by analyzing the reference species listed in samples and reference strain. As Fig. 3 shown, in the melting curve, the T_m_ value of standard strain *M. pneumoniae* M129 (ATCC 29342) that amplified by ASPCR was 79.4, and the T_m_ of the other reference strains were as following: *Neisseria mucus* (T_m_ = 87.92), *Klebsiella pneumoniae* (T_m_ = 86.83), *Escherichia coli* (T_m_ = 84.29), *Staphylococcus aureus* (T_m_ = 84.84), *Streptococcus pneumoniae* (T_m_ = 85.56), *Pseudomonas aeruginosa* (T_m_ = 88.82), *Mycoplasma fermentans* (T_m_ = 84.84), *Ureaplasma urealyticum* (T_m_ = 85.38), *Mycoplasma pyriformis* (T_m_ = 84.65), *Mycoplasma hominis* (T_m_ = 85). All the above indicated that by amplifying the Ct value of the curve and the Tm value of the melting curve, the DNA of *M. pneumoniae* could be successfully distinguished from other strains’ DNA.

### Sensitivity and accuracy of ASPCR

**Fig. 4:**
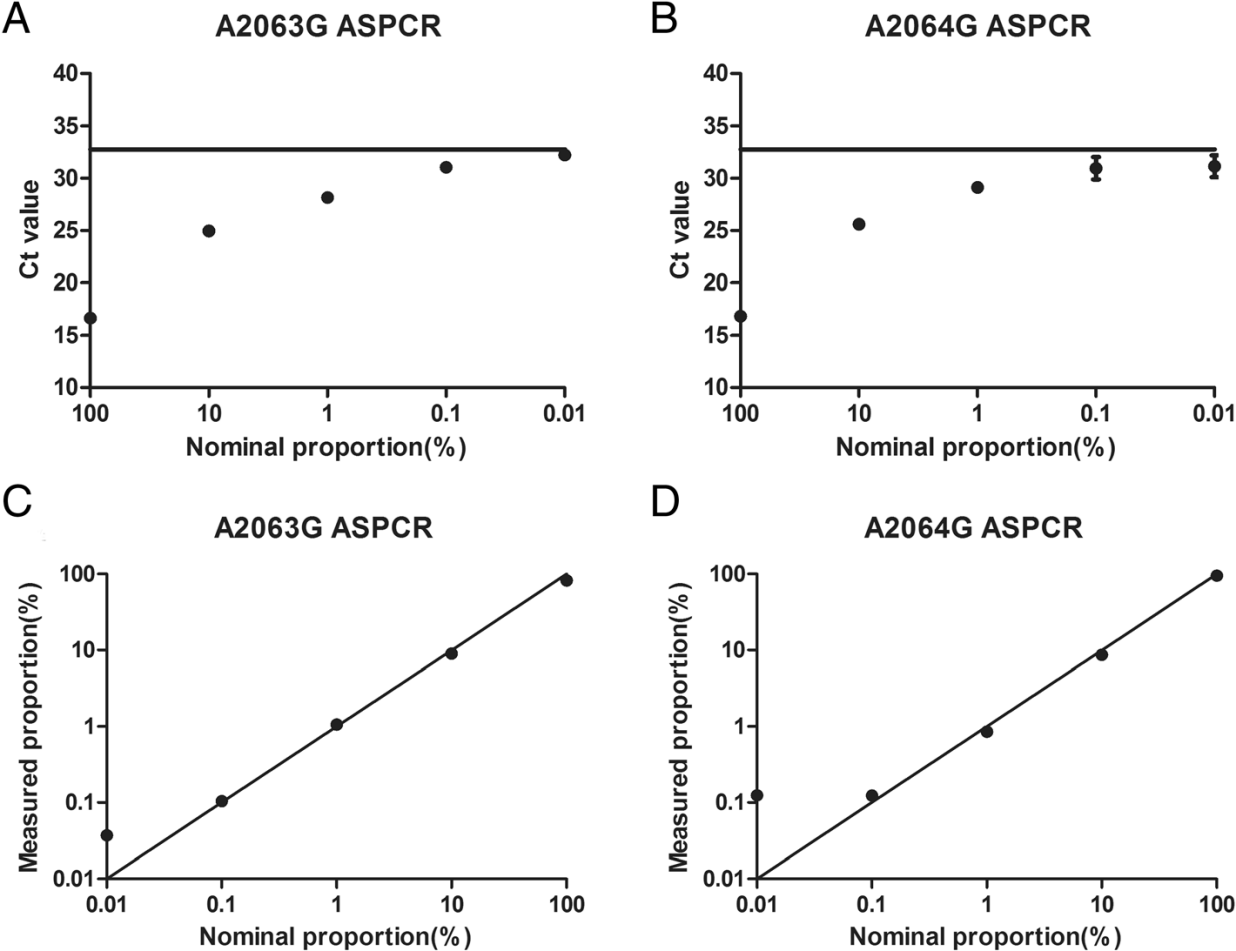
Sensitivity and accuracy of A2063G and A2064G. **A-B**: The sensitivity of A2063G and A2064G. Transverse line: The cut-off value line of each mutation. The cut-off value was defined as the mean Ct value plus three standard deviations (SD) of 12 independent determinations of 10^5^ copies wild-type template with mutant-specific primer. The Ct values of the mixtures with mutant template ranging from 0.01 to 100% were less than the cut-off values of detecting A2063G and A2064G assays. These indicated that resistance-associated mutations could still be detected until their proportion was about 0.01%. **C-D**: The accuracy of A2063G and A2064G. Measured proportion = Measured mutant copy number/ measured total DNA copy number× 100%. Measured and nominal proportions were comparable. The measured proportions of detecting A2063G and A2064G assays were accurate down to 0.1%

The cut-off Ct value were 33.57 and 32.76 for detecting of A2063G and A2064G, respectively. The Ct values of the mixtures with mutant template ranging from 0.01 to 100% were less than the cut-off values of detecting A2063G and A2064G assays (Fig. 4). These indicated that resistance-associated mutations could still be detected until their proportion was about 0.01%. The Ct values of mixtures amplified with corresponding specific and non-specific primer were interpolated into the corresponding standard curves to get the numbers of mutant template and the total template. Then the proportions of mutant template in the mixtures were calculated, and the measured and nominal proportions were comparable (Fig. 4). The measured proportions of detecting A2063G and A2064G assays were accurate down to 0.1%. The sensitivities of ASPCR testing A2063G and A2064G were both determined as 0.1%, considering the cut-off value and accuracy of these assays.

### Reproducibility of ASPCR

The intra-assay CVs of detecting A2063G and A2064G assays were below 0.11, and the inter-assay CVs were below 0.18 (Table 2). These results indicated that the ASPCR assays for detecting A2063G and A2064G had good reproducibility.

**Table 2:** Intra-assay and inter-assay coefficients of variation (CVs)

Mutant proportion (%)	CV Intra-assay		CV Inter-assay	
	A2063G	A2064G	A2063G	A2064G
100	0.050	0.065	0.180	0.045
10	0.054	0.093	0.168	0.045
1	0.052	0.104	0.147	0.025

### Prevalence of M. pneumoniae and macrolide-resistant genotype

For 178 clinical samples, 164 samples were found to be *M. pneumoniae* positive by ASPCR. Among the 164 samples, 90.85% (149/164) samples were found to have the resistance mutations including 61.07% (91/149) with A2063G, 3.36% (5/149) with A2064G and 35.57% (53/149) with both mutations, while only 9.15% (15/164) of samples contained the wild-type (Table 3). Moreover, among the 149 samples, the mixing genotypes of A2063G and A2064G, A2063G and wild-type, A2064G and wild-type, and A2063G, A2064G and wild-type were detected in 10, 53, 5 and 43 samples, separately (Table 3). The clinical sample detection results of ASPCR indicated that the sensitive and resistant quasispecies were co-existed in most (67.79%, 101/149) of the *M. pneumoniae* positive samples.

**Table 3:** Comparison of the performance characteristics of two methods for detection of macrolide resistance mutations at 23S rRNA

Genotypes	Method	
	ASPCR	Nested PCR + sequencing
Total	178	178
*M. pneumoniae* positive (no, %)	164	153
Resistance mutations	149	110
A2063G	38	109
A2063G + WT	53	0
A2064G + WT	5	0
A2063G + A2064G + WT	43	0
A2063G + A2064G	10	0
A2064G	0	1
WT	15	43
Negative	14	25

WT (wild-type): no macrolide resistance mutation detected

The results of detecting the 178 clinical samples using ASPCR were compared with the results of detection by nested PCR with sequencing. The *M. pneumoniae* positive ratios of ASPCR and nested PCR with sequencing were 92.13% (164/178) and 85.96% (153/178), respectively. A relatively low coincidence ratio (kappa coefficient = 0.515) of detecting *M. pneumoniae* infection was shown between the ASPCR assays and nested PCR with sequencing analysis. The drug-resistance ratios of these samples tested by ASPCR and nested PCR with sequencing were 90.85% (149/164) vs 71.90% (110/153) (χ^2^ = 19.031, *P* < 0.001)**.** The positive ratios of drug-resistant associated mutations tested by ASPCR and nested PCR with sequencing were as follows: A2063G, 87.80% (144/164) vs. 71.24% (109/153) (χ^2^ = 13.476, *P* < 0.001), A2064G, 35.37% (58/164) vs. 0.65% (1/153) (χ^2^ = 59.320, *P* < 0.001) (Table 3). The prevalence rates of A2063G and A2064G tested by ASPCR were significantly higher than the results of nested PCR with sequencing.

The distributions of cases with different proportions of A2063G and A2064G detected by ASPCR were analyzed and compared with general sequencing after nested PCR. The frequencies of the A2063G were < 30% in 28 specimens, 30 to 50% in 24 specimens, 50 to 100% in 92 specimens. The frequencies of the A2064G were < 30% in 56 specimens, 30 to 50% in 2 specimens, while the 50 to 100% frequency of A2064G was not detected in the clinical specimens (Table 4). The cases carrying A2063G and A2064G tested by nested PCR with sequencing were clustered significantly on higher (≧30 and < 50% and≧50%) mutation proportions determined by ASPCR. These results indicated that the sensitivity of ASPCR was significantly higher than that of the nested PCR with sequencing.

**Table 4:** Distribution of cases on different proportions of A2063G and A2064G tested by ASPCR and compared with nested PCR following with sequencing

Method		Proportion of mutation (ASPCR) (%)						Total
		<S	≧S and < 30	≧30 and < 50	≧50 and < 100	100	Negative	
Nested PCR + sequencing	A2063G	5	14	13	41	33	3	109
	WT	10	12	9	12	1	0	44
	Negative	5	2	2	1	4	11	25
Total		20	28	24	54	38	14	178
Nested PCR + sequencing	A2064G	0	0	1	0	0	0	1
	WT	93	55	1	0	0	3	152
	Negative	13	1	0	0	0	11	25
Total		106	56	2	0	0	14	178

S: the sensitivity of the ASPCR assay

Because of the information limitation of the clinical specimens, the general characteristics for only 154 patients with infection by wild-type and mutant *M. pneumoniae* determined by ASPCR were compared (Table 5). There was no significant difference in median age, gender and disease process among these groups analyzed in different analysis (*P* > 0.05 for all comparisons). An alternative analysis was conducted by allocating infected persons based on the percentage of the mutant *M. pneumoniae* determined by ASPCR (Table 5), and there was no significant differences between the three groups of WT, low frequency mutant group and high frequency mutant group for all parameters (P > 0.05 for all comparisons).

**Table 5:** Comparison of characteristics of patients infected by WT and different frequency mutant *M. pneumoniae* determined by ASPCR assays

Characteristic	WT	Mutant *M. pneumoniae* (Low frequency/High frequency)	*P* Two groups/Three groups
n	15	139 (52/87)	
Mean age (year)	5.58 ± 3.35	6.43 ± 3.14 (6.38 ± 2.91/6.46 ± 3.29)	0.408/0.646 (*F* = 0.438)
Female (no, %)	7 (46.7%)	64 (46.0%) (26 (40.6%)/38 (59.4%))	0.963/0.769 (χ^2^ = 0.526)
Disease process (days)	6.86 ± 7.58	8.26 ± 6.49 (9.42 ± 8.79/7.71 ± 5.05)	0.698/0.336 (*F* = 1.099)

WT: no macrolide resistance mutation detected (group A); mutant *M. pneumoniae*: A2063G or A2064G mutation detected (group B); low frequency: macrolide resistance mutation present at low percentage (≧S and < 50%) (group C); high frequency: macrolide resistance mutation present at high percentage (≧50%) (group D); two groups: WT (group A) and mutant *M. pneumoniae* (group B); three groups: WT (group A), low frequency (group C) and high frequency (group D)

## Discussion

Comparing with the routine drug resistance detecting assays, ASPCR is a highly sensitive, accurate, time-saving and high throughput method for detecting resistant *M. pneumoniae* and analyzing the mutation frequency. The sensitivity and accuracy of ASPCR were directly proportional to the discrimination ability of the specific primers, which was strongly influenced by the particular 3′ end base sequence. The bases near the 3’end of the specific primer were replaced by hypoxanthine in order to enhance the specificity of the ASPCR assays [27]. The number and location of hypoxanthine were critical for the discrimination ability of the specific primer. In this study, each ASPCR assay could detect more than 10 copies/reaction of 23S rRNA gene and the presence of mutant species with the sensitivity down to 0.1%. Although the mismatch occurred at the 3’end of the specific primer, the measured proportions of detecting A2063G and A2064G assays were accurate down to 0.1%, and the intra-assay and inter-assay CVs of the ASPCR were below 0.18.

The main limitation of the ASPCR is that only one mutation can be detected in every reaction, while many mutations can be tested at once using nested PCR with sequencing. Prior study had demonstrated that the polymorphisms that occur in the specific primer binding sites can significantly impair the accuracy of ASPCR assays [27]. The polymorphism must be taken into account when testing clinical samples and the prior sequencing is suggested to overcome this limitation, while ASPCR was applied in detecting the drug-resistance associated mutations of HIV [31]. The polymorphism had little effect on the detection of A2063G and A2064G mutations of *M. pneumoniae*, because of the lower variability of *M. pneumoniae* compared with HIV and the high conserved sequence near these mutations. Considering this, it is more suitable to apply ASPCR detecting mutations of *M. pneumoniae* than that of other hypervariable virus.

Previous, Chan et al. [32] compared the detecting result of low-frequency MRMP quasispecies that obtained by pyrosequencing with those obtained by Sanger sequencing and SimpleProbe PCR coupled to melting curve analysis on respiratory specimens. The results indicated that pyrosequencing identified A2063G MRMP quasispecies populations in 78.8% (67/88) of the specimens, and only 38.8% (26/67) of these specimens with the A2063G quasispecies detected by pyrosequencing were found to be A2063G quasispecies by Sanger sequencing or SimpleProbe PCR. In this study, the results showed 96.64% of the resistant specimens had A2063G mutation, 35.57% (53/149) with both A2063G and A2064G mutations that was not tested by other method in previous study, such as Chan et al. [32], Lin et al. [19] and Ji et al. [21], which might indicate ASPCR is a highly sensitive, accurate, time-saving and high throughput method for detecting resistant *M. pneumoniae*. Of the 164 *M. pneumoniae* positive samples, 61.59% had the mixing of wild-type and drug resistant *M. pneumoniae*, and 56.44% of the latter contained the drug resistance mutations at low frequency (≤50%). These results of ASPCR indicated that sensitive and resistant quasispecies coexisted in most of the *M. pneumoniae*-positive samples, and the resistant mutations could be at a relative low frequency. These finds were important directive significance for the clinical management. Furthermore, compared to the nested PCR with sequencing, ASPCR testing has short the turnaround time, is highly sensitive for testing *M. pneumoniae* and is able to discriminate the samples with resistant *M. pneumoniae* at a very low frequency*.* All these make the ASPCR an attractive method for the highly sensitive and rapid diagnosis of *M. pneumoniae.* The study of minor resistant variants in *M. pneumoniae* infection is relevant to understanding the mechanisms of the generation and development of drug resistance. It is also very important for clinical management to test and monitor the drug-resistance associated mutations.

There are several limitations to our study. Firstly, the sample size was small. Secondly, clinical sample information was incomplete collection, which may lead to inaccurate statistical analysis. Thirdly, the specificity of the ASPCR assay was only used to test on the strains that associated with respiratory infections, not on respiratory tract specimens. Therefore, in future, a large sample size with complete clinical data is needed to further study to confirm the study, and the related factors between *M. pneumoniae* resistance and infection are needed to analyze.

## Conclusions

In generally, due to its highly sensitivity and accuracy, the ASPCR assays can be used as a particularly useful tool for resistance surveillance and studying the mechanism of the generation and development of drug resistance. In future study, the ASPCR assays can be used to characterize the dynamics of mutants in vivo by measuring the proportion of A2063G and A2064G variants in serial pharyngeal swabs specimensying and study the kinetics of selection and decay of point resistance mutations. For future application, ASPCR can also be easily developed for detecting other mutations associated with drug resistance of *M. pneumoniae* using proper standards and primers.

## Data Availability

The datasets generated and/or analysed during the current study are not publicly available due to provisions for the preservation of relevant raw data in our laboratory, but are available from the corresponding author on reasonable request.

## Abbreviations

ASPCR: allele-specific real-time PCR
CVs: coefficient of variations
*M. pneumoniae*: *Mycoplasma pneumoniae*
MIC: minimum inhibitory concentration
MRMP: macrolide-resistant *M. pneumoniae*
NTC: no template control
RFLP: restriction fragment length polymorphism
SD: standard deviations
